# Neutrophil-to-Lymphocyte Ratio as a Predictive Biomarker for Stroke Severity and Short-Term Prognosis in Acute Ischemic Stroke With Intracranial Atherosclerotic Stenosis

**DOI:** 10.3389/fneur.2021.705949

**Published:** 2021-07-29

**Authors:** Yuanlin Ying, Fang Yu, Yunfang Luo, Xianjing Feng, Di Liao, Minping Wei, Xi Li, Qin Huang, Zeyu Liu, Lin Zhang, Tingting Zhao, Ruxin Tu, Jian Xia

**Affiliations:** ^1^Department of Neurology, Xiangya Hospital, Central South University, Changsha, China; ^2^Clinical Research Center for Cerebrovascular Disease of Hunan Province, Central South University, Changsha, China; ^3^National Clinical Research Center for Geriatric Disorders, Xiangya Hospital, Central South University, Changsha, China

**Keywords:** ischemic stroke, intracranial atherosclerotic stenosis, neutrophil-to-lymphocyte ratio, stroke severity, short-term prognosis

## Abstract

**Background:** Neutrophil-to-lymphocyte ratio (NLR) is an indicator of poor prognosis in acute ischemic stroke (AIS), but associations between NLR with stroke severity and prognosis of intracranial atherosclerotic stenosis (ICAS)-related ischemic events have not been well-elucidated; therefore, we aimed to evaluate whether admission NLR levels correlate with the early stroke severity and short-term functional prognosis in patients with symptomatic intracranial atherosclerotic stenosis (sICAS).

**Methods:** This retrospective study enrolled 899 consecutive patients with AIS attributed to ICAS at Xiangya Hospital stroke center between May 2016 and September 2020. The initial stroke severity was rated by the admission National Institutes of Health Stroke Scale (NIHSS) scores, and the short-term prognosis was evaluated using the 14-day modified Rankin Scale (mRS) scores after stroke onset. A severe stroke was defined as NIHSS >8; an unfavorable functional outcome was defined as mRS scores of 3–6. Admission NLR was determined based on circulating neutrophil and lymphocyte counts.

**Results:** The median admission NLR of all patients was 2.80 [interquartile range (IQR), 2.00–4.00]. In univariate analysis, admission NLR was significantly elevated in patients with severe stroke and poor short-term prognosis. After multivariate adjustment, admission NLR levels were significantly correlated with severe stroke [odds ratio (OR), 1.132; 95% confidence interval (95% CI), 1.038–1.234; *P* = 0.005] and unfavorable short-term prognosis (OR, 1.102; 95% CI, 1.017–1.195; *P* = 0.018) in Model 1. In Model 2, the highest NLR tertile (≥3.533) remained an independent predictor of severe stroke (OR, 2.736; 95% CI, 1.590–4.708; *P* < 0.001) and unfavorable functional outcome (OR, 2.165; 95% CI, 1.416–3.311; *P* < 0.001) compared with the lowest NLR tertile (<2.231). The receiver operating characteristic (ROC) curves showed the predictability of NLR regarding the stroke severity [area under the curve (AUC), 0.659; 95% CI, 0.615–0.703; *P* < 0.001] and short-term prognosis (AUC, 0.613; 95% CI, 0.575–0.650; *P* < 0.001). The nomograms were constructed to create the predictive models of the severity and short-term outcome of sICAS.

**Conclusions:** Elevated admission NLR levels were independently associated with the initial stroke severity and could be an early predictor of severity and poor short-term prognosis in AIS patients with ICAS, which might help us identify a target group timely for preventive therapies.

## Introduction

Stroke is a growing public health concern and remains a leading cause of mortality and disability worldwide (1). Of all different stroke subtypes, ischemic stroke is the most common subtype, accounting for about 80% of all strokes and mainly caused by large artery atherosclerosis (LAA), especially intracranial atherosclerotic stenosis (ICAS), which has a higher prevalence in Asians, Africans, and Hispanics compared with Caucasians (1–3). Accumulating evidence suggests that ICAS was associated with increased risks of cognitive impairment, dementia, stroke recurrence, and death (4–6). However, the pathogenesis of ICAS has not been well-investigated. Previous studies have established that atherosclerosis is an inflammatory disorder mediated by activation of inflammatory markers and infiltration of various inflammatory cells, especially activated macrophages and T cells, which could release metalloproteinases, thus leading to atherosclerotic plaque instability or rupture, ischemia, and infarction (7, 8). Since ICAS is on the spectrum of atherosclerosis, inflammation might also be a pathophysiological mechanism for the development and progression of ICAS.

Neutrophil-to-lymphocyte ratio (NLR) is emerging as an objective and easily accessible indicator of systemic inflammatory status, reflecting the balance between neutrophils and lymphocytes in peripheral blood (9). An increase in NLR levels has been reported to be associated with atherosclerotic events, serving as a prognostic predictor in coronary artery disease (CAD), peripheral arterial occlusive disease (PAOD), and ischemic stroke (10–12). Specifically, it has previously been observed that high NLR levels were correlated with stroke severity, poor functional outcomes, and recurrent ischemic events in stroke patients (12, 13). Furthermore, large vessel occlusion patients with high admission NLR values had increased risks of symptomatic intracranial hemorrhage (sICH) and 3-month mortality after mechanical thrombectomy (14). Even in a healthy population, an elevated NLR has been proved to be linked to the prevalence and burdens of ICAS (15). However, few studies have examined the association between NLR and symptomatic intracranial atherosclerotic stenosis (sICAS). Although a recent study has explored the relationships between NLR with ICAS and ischemic stroke and demonstrated that the association between NLR and ischemic stroke was partially mediated by ICAS (16), there is a relative paucity of studies investigating the underlying relationships between admission NLR with the initial severity and prognosis of sICAS.

In this study, we aimed to determine the potential predictive capacity of admission NLR levels for stroke severity and short-term prognosis of patients with acute ischemic stroke (AIS) attributed to ICAS to help identify a promising therapeutic strategy.

## Materials and Methods

### Patients and Population

This study retrospectively enrolled and analyzed 899 consecutive patients with AIS attributed to ICAS, who were admitted to Xiangya Hospital stroke center between May 2016 and September 2020, satisfying the following inclusion criteria: (1) age ≥18 years; (2) time interval from symptom onset to admission ≤72 h; and (3) blood sampling performed within 24 h after hospital admission. Those patients, who had cardioembolic stroke or evidence of cardioembolic propensity, severe stenosis of extracranial carotid artery, malignant tumors, severe renal or hepatic diseases, hematological diseases, inflammatory or infectious diseases, or history of immunosuppressant medications within the past 3 months, were excluded from the current study. The criteria for diagnosing AIS have been described in detail previously (17). Each participant in this study has provided the informed consent form. The study was approved by the Medical Ethics Committee of Xiangya Hospital, Central South University, China.

### Stroke Severity and Outcome Assessment

The National Institutes of Health Stroke Scale (NIHSS) scores have been collected on admission for the assessment of initial stroke severity, stratifying patients into two groups: mild stroke (NIHSS score ≤8) and severe stroke (NIHSS score >8). The 14-day modified Rankin Scale (mRS) scores after stroke onset have been used to evaluate the short-term prognosis after stroke, and patients were therefore categorized into two groups: favorable functional outcome group with mRS scores of 0–2 and unfavorable functional outcome group with mRS scores of 3–6.

### Clinical and Laboratory Data Collection

Baseline characteristics including demographics, cardiovascular risk factors, clinical data, and laboratory data were collected and recorded on admission. Histories of hypertension, diabetes mellitus, dyslipidemia, and coronary artery disease (CAD), as well as cigarette smoking and alcohol intake, have been considered cardiovascular risk factors. Besides, systolic and diastolic blood pressure levels were measured on admission. Admission NIHSS scores and 14-day mRS scores were collected as previously mentioned. The treatments including intravenous thrombolysis (IVT), endovascular treatment (EVT), and antiplatelet therapy were collected during hospitalization. Blood samples were collected within 24 h after admission for the examination of laboratory parameters, including white blood cell (WBC), platelet (Plt), neutrophil, lymphocyte, NLR, fasting blood glucose (FBG), uric acid (UA), homocysteine (Hcy), fibrinogen (Fib), and a lipid profile which consists of total cholesterol (TC), triglyceride (TG), high-density lipoprotein (HDL), and low-density lipoprotein (LDL). The diagnostic criteria and parameters have been described in detail in our prior work (17, 18). Admission NLR was calculated based on neutrophil and lymphocyte counts in the peripheral blood.

### Radiological Assessment

Standard magnetic resonance imaging (MRI) of the brain was performed in all participants on 1.5 or 3.0 T scanners, including sequences of T1-weighted, T2-weighted, fluid-attenuated inversion recovery (FLAIR), and diffusion-weighted imaging (DWI). The lesions of stenosis or occlusion were measured using three-dimensional time-of-flight (3D TOF) magnetic resonance angiography (MRA), computerized tomography angiography (CTA), or digital subtraction angiography (DSA). The presence of ICAS was defined as stenosis or occlusion of intracranial atherosclerotic arteries rated by two experienced radiologists, and the percentage of luminal stenosis was calculated according to the Warfarin-Aspirin Symptomatic Intracranial Disease (WASID) criteria, classifying the severity of stenosis into mild (<50%), moderate (50–69%), severe (70–99%), and occlusion (100%) (19). Moreover, hemorrhagic transformations (HT) and cerebral edema (CED) were defined using the computed tomography (CT) or MRI and classified according to the European Cooperative Acute Stroke Study-2 (ECASS-II) criteria by at least two experienced radiologists (20, 21). In this study, we stratified HT into hemorrhagic infarction (HI) 1, HI2, parenchymal hemorrhage (PH) 1, and PH2; and we dichotomized CED into none or mild (grades 0–1) and moderate-to-severe (grades 2–3) (20, 22).

### Statistical Analysis

Continuous variables were presented as mean with standard deviation (SD) or median with interquartile range (IQR) for data with normal and non-normal distributions, respectively, whereas categorical variables were described as number and percentage. For continuous variables, Student *t*-test and Mann-Whitney *U*-test were used for intergroup comparisons, while for categorical variables, Pearson χ^2^-test was used to assess the intergroup difference. Multivariate binary logistic regression analysis was conducted to evaluate associations between NLR with early severity and short-term prognosis after adjustment for age, sex, and other possible confounders, involving all variates with a *P* < 0.05 in initial univariate analysis, with results exhibited as odds ratios (OR) and 95% confidence interval (CI). Model 1 (the laboratory parameters as continuous variables) and Model 2 (the laboratory parameters as dichotomous or trichotomous variables) were used in multivariate binary logistic regression analysis, and NLR levels were categorized into tertiles: the first tertile, <2.231; the second tertile, 2.231–3.533; and the third tertile, ≥3.533 in Model 2. To evaluate the predictive capacity of NLR to discriminate early severity and short-term prognosis, we illustrated receiver operating characteristic (ROC) curves, measured the area under the curve (AUC), and determined the CIs. The nomograms were plotted using the independent significant variables based on the results of multivariate logistic regression analysis. All statistical analyses were conducted with Empower Stats (http://www.empowerstats.com) and R software (http://www.R-project.org/). *P* < 0.05 was considered statistically significant.

## Results

### Patient Baseline Characteristics

**Figure 1:**
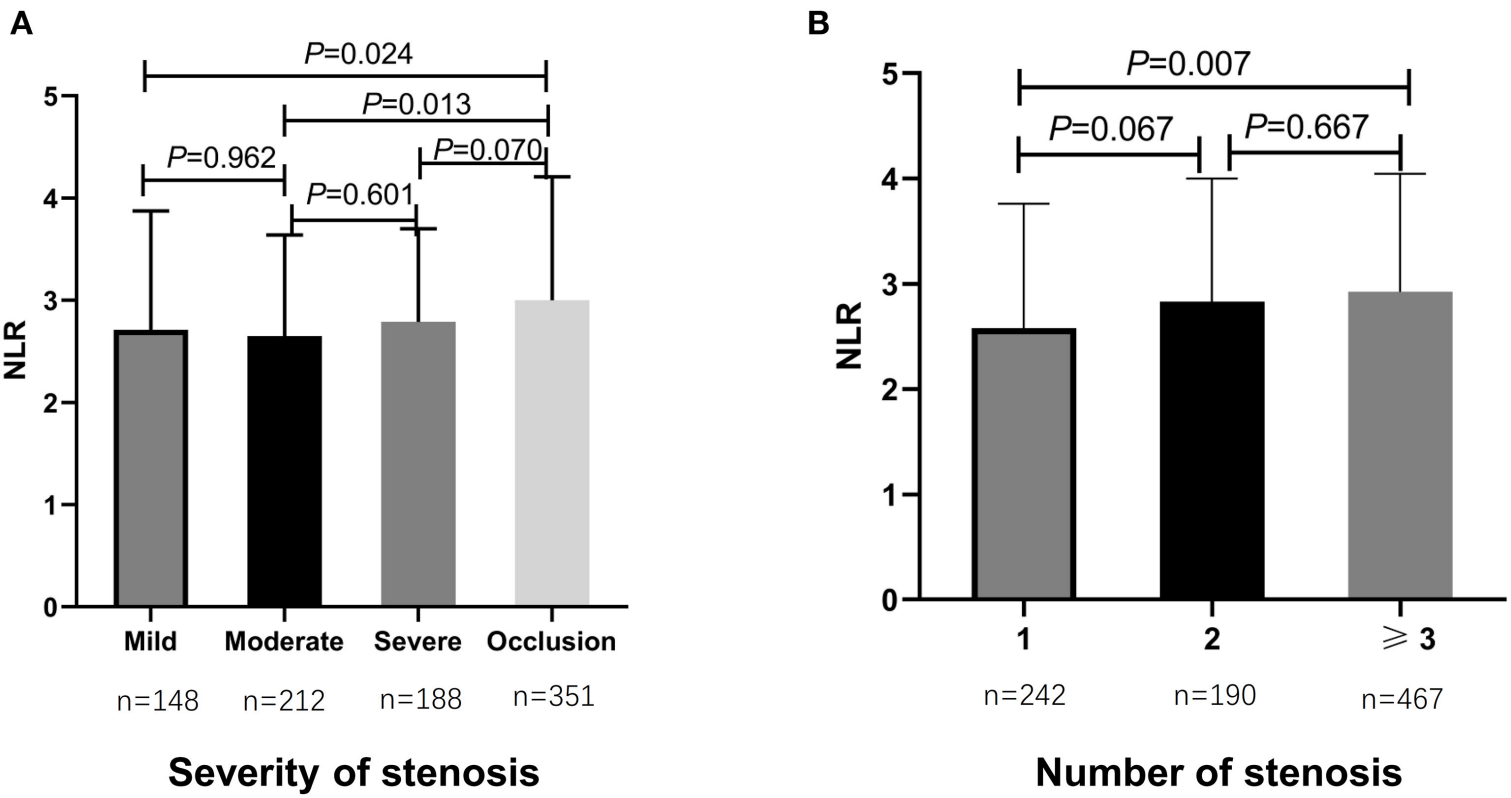
Comparisons of admission NLR levels according to the severity and number of intracranial atherosclerotic stenosis in sICAS patients. **(A)** Comparison of admission NLR levels according to the severity of intracranial atherosclerotic stenosis. **(B)** Comparison of admission NLR levels according to the number of intracranial atherosclerotic stenosis. *P* < 0.05 was considered statistically significant. NLR, neutrophil-to-lymphocyte ratio; sICAS, symptomatic intracranial atherosclerotic stenosis.

**Figure 2:**
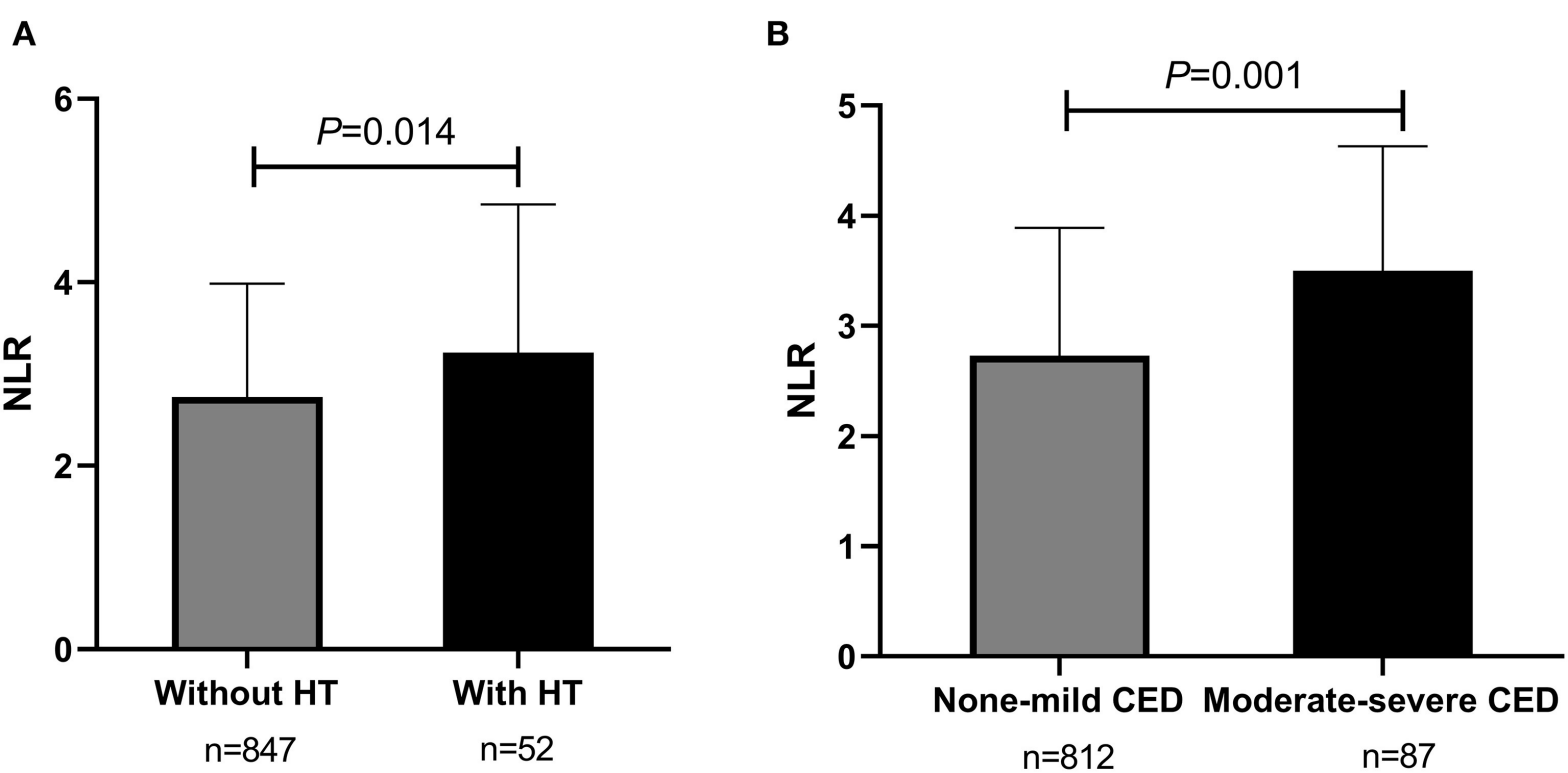
Comparisons of admission NLR levels according to HT and CED in sICAS patients. **(A)** Comparison of admission NLR levels according to HT (without HT vs. with HT). **(B)** Comparison of admission NLR levels according to CED (none-mild CED vs. moderate-severe CED). *P* < 0.05 was considered statistically significant. NLR, neutrophil-to-lymphocyte ratio; sICAS, symptomatic intracranial atherosclerotic stenosis; HT, hemorrhagic transformation; CED, cerebral edema.

From May 2016 to September 2020, totally 899 consecutive AIS patients with ICAS were enrolled in the study analysis. The median age of all participants was 61 years (IQR, 53–68 years) and 64.3% (*n* = 578) were male (as shown in Table 1). Cardiovascular risk factors included histories of hypertension (*n* = 699, 77.8%), diabetes mellitus (*n* = 310, 34.5%), dyslipidemia (*n* = 412, 45.8%), CAD (*n* = 130, 14.5%), smoking (*n* = 390, 43.4%), and drinking (*n* = 302, 33.6%) (as shown in Table 1). During hospitalization, almost all patients were on antiplatelet therapy, in which 48.9% (*n* = 440) were using mono-antiplatelet drugs, and 48.2% (*n* = 433) were using dual antiplatelet drugs (as shown in Table 1). The rates of participants treated with IVT and EVT were 2.3% (*n* = 21) and 2.8% (*n* = 25), respectively (Table 1). Additionally, patients were stratified as having mild stenosis (*n* = 148, 16.5%), moderate stenosis (*n* = 212, 23.6%), severe stenosis (*n* = 188, 20.9%), and occlusion (*n* = 351, 39.0%) (Table 1). The median admission NLR of all included patients was 2.80 (IQR, 2.00–4.00) (Table 1). Patients with intracranial atherosclerotic artery occlusion had statistically higher admission NLR levels compared to patients with mild stenosis (*P* = 0.024) and moderate stenosis (*P* = 0.013) (as shown in Figure 1A). Regarding the number of stenosis, higher admission NLR levels were documented in patients with multiple intracranial artery stenosis (the number of stenosis ≥3) (*n* = 467, 51.9%) compared to patients with single stenosis (*n* = 242, 26.9%; *P* = 0.007) (as shown in Table 1 and Figure 1B). Besides, patients with HT (*n* = 52, 5.8%) had statistically higher admission NLR levels compared to patients without HT (*P* = 0.014), and patients with moderate-severe CED (*n* = 87, 9.7%) had statistically elevated admission NLR levels compared to patients with none-mild CED (*P* = 0.001) (as shown in Table 1 and Figure 2). The median admission NIHSS score of the participants was 5 (IQR, 2–8), and the initial stroke severity was stratified according to admission NIHSS scores as follows: mild stroke, NIHSS score ≤8 (*n* = 698, 77.6%); severe stroke, NIHSS score >8 (*n* = 201, 22.4%) (as shown in Table 1). The median mRS score on the 14^th^ day after stroke onset of the participants was 2 (IQR, 1–3), and the short-term prognosis was stratified by 14-day mRS scores as follows: a favorable outcome, mRS scores ≤2 (*n* = 521, 58.0%); an unfavorable outcome, mRS scores >2 (*n* = 378, 42.0%) (as shown in Table 1). Baseline clinical characteristics, as well as the admission laboratory data of the participants, were summarized in Table 1.

**Table 1:** Baseline characteristics of the study population.

**Variables**	**Patients (*n* = 899)**
Age (years)	61 (53–68)
Sex, male, *N* (%)	578 (64.3%)
Hypertension, *N* (%)	699 (77.8%)
Diabetes, *N* (%)	310 (34.5%)
Dyslipidemia, *N* (%)	412 (45.8%)
CAD, *N* (%)	130 (14.5%)
Smoking, *N* (%)	390 (43.4%)
Drinking, *N* (%)	302 (33.6%)
IVT, *N* (%)	21 (2.3%)
EVT, *N* (%)	25 (2.8%)
**Antiplatelet drugs**, ***N*****(%)**	
Mono-antiplatelet	440 (48.9%)
Dual antiplatelets	433 (48.2%)
None	26 (2.9%)
**Severity of stenosis**, ***N*****(%)**	
Mild	148 (16.5%)
Moderate	212 (23.6%)
Severe	188 (20.9%)
Occlusion	351 (39.0%)
**Number of stenosis**, ***N*****(%)**	
1	242 (26.9%)
2	190 (21.1%)
≥3	467 (51.9%)
HT, *N* (%)	52 (5.8%)
**ECASS II classification**, ***N*****(%)**	
HI1	12 (1.3%)
HI2	19 (2.1%)
PH1	10 (1.1%)
PH2	11 (1.2%)
Moderate–severe CED, *N* (%)	87 (9.7%)
NIHSS score	5 (2–8)
NIHSS ≤ 8, *N* (%)	698 (77.6%)
NIHSS > 8, *N* (%)	201 (22.4%)
mRS score	2 (1–3)
mRS 0–2, *N* (%)	521 (58.0%)
mRS 3–6, *N* (%)	378 (42.0%)
SBP (mmHg)	144.00 (132.00–158.00)
DBP (mmHg)	84.00 (76.00–93.00)
WBC (×10^9^/L)	6.90 (5.60–8.50)
Plt (×10^9^/L)	207.00 (169.00–251.00)
Neutrophil (×10^9^/L)	4.40 (3.40–5.90)
Lymphocyte (×10^9^/L)	1.60 (1.20–2.00)
NLR	2.80 (2.00–4.00)
TC (mmol/L)	4.37 (3.58–5.18)
TG (mmol/L)	1.56 (1.18–2.12)
HDL (mmol/L)	1.00 (0.86–1.20)
LDL (mmol/L)	2.70 (2.12–3.32)
FBG (mmol/L)	5.69 (4.96–7.29)
UA (μmol/L)	311.20 (253.10–378.20)
Hcy (μmol/L)	13.36 (11.06–16.42)
Fib (g/L)	3.28 (2.76–4.03)

*CAD, coronary artery disease; IVT, intravenous thrombolysis; EVT, endovascular therapy; HT, hemorrhagic transformation; ECASS II, European Cooperative Acute Stroke Study II; HI, hemorrhagic infarction; PH, parenchymal hematoma; CED, cerebral edema; NIHSS, the National Institutes of Health Stroke Scale; mRS, modified Rankin Scale; SBP, systolic blood pressure; DBP, diastolic blood pressure; WBC, white blood cell; Plt, platelet; NLR, neutrophil-to-lymphocyte ratio; TC, total cholesterol; TG, triglyceride; HDL, high-density lipoprotein; LDL, low-density lipoprotein; FBG, fasting blood glucose; UA, uric acid; Hcy, homocysteine; Fib, fibrinogen*.

### Association Between NLR and Initial Stroke Severity in sICAS Patients

**Figure 3:**
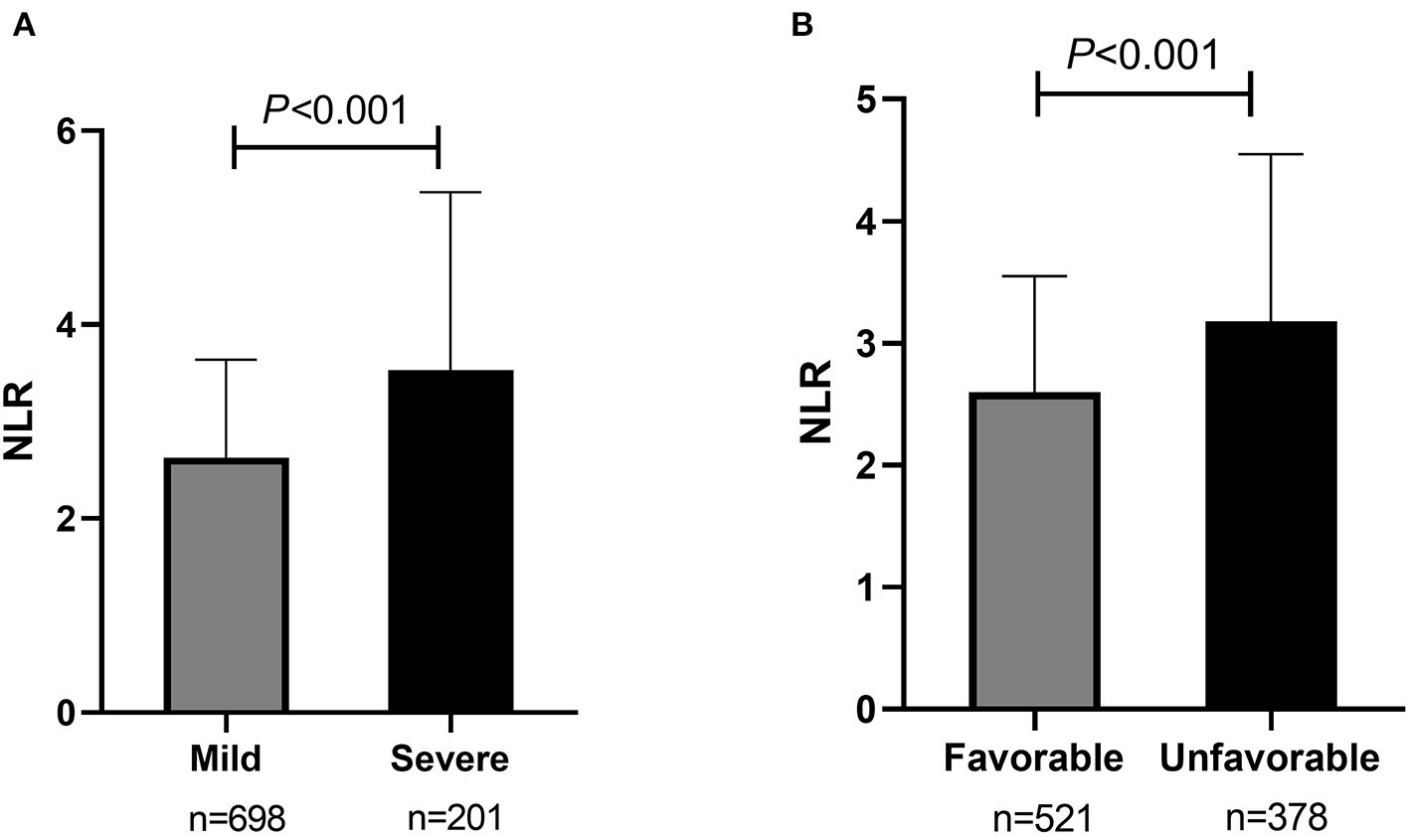
Comparisons of admission NLR levels according to the initial stroke severity and short-term prognosis of sICAS. **(A)** Comparison of admission NLR levels according to the initial stroke severity (severe stroke vs. mild controls). **(B)** Comparison of admission NLR levels according to the short-term prognosis (unfavorable outcome vs. favorable outcome). NLR, neutrophil-to-lymphocyte ratio; sICAS, symptomatic intracranial atherosclerotic stenosis.

Of all participants, 698 (77.6%) were classified as having a mild stroke, and 201 (22.4%) had a severe stroke as mentioned before. In univariate analysis, sex, dual antiplatelet therapy, stenosis severity, moderate-severe CED, WBC, neutrophils, lymphocytes, NLR, FBG, UA, and Fib were significantly related to stroke severity at admission with a *P* < 0.05 (as shown in Table 2). Patients with severe stroke had statistically elevated admission NLR levels (median, 3.53; IQR, 2.43–5.25) compared to patients with mild stroke (median, 2.63; IQR, 1.93–3.64; *P* < 0.001) (as shown in Table 2 and Figure 3A). Moreover, the proportions of female gender, occlusion, and moderate-severe CED, and levels of WBC, neutrophil, FBG, and Fib in the severe stroke group were higher than those in the mild stroke group, whereas the proportion of dual antiplatelet therapy and the levels of lymphocyte and UA were lower in the severe stroke group (as shown in Table 2). Multivariate logistic regression analysis indicated that admission NLR levels were significantly correlated with severe stroke (OR, 1.132; 95% CI, 1.038–1.234; *P* = 0.005) in Model 1 and both the middle and the highest NLR tertiles were significantly correlated with severe stroke (tertile 2: OR, 1.851; 95% CI, 1.089–3.145; *P* = 0.023; and tertile 3: OR, 2.736; 95% CI, 1.590–4.708; *P* < 0.001) compared with the lowest NLR tertile in Model 2 after adjustment for age, sex, dual antiplatelet therapy, stenosis severity, moderate-severe CED, WBC, FBG, UA, and Fib (as shown in Table 3).

**Table 2:** Clinical characteristics of sICAS patients according to the initial stroke severity and short-term prognosis.

**Variables**	**Mild (*n* = 698)**	**Severe (*n* = 201)**	***P-*value**	**Favorable (*n* =521)**	**Unfavorable (*n* = 378)**	***P-*value**
	**(NIHSS ≤ 8)**	**(NIHSS >8)**		**(mRS 0–2)**	**(mRS 3–6)**	
Age (years)	61 (52–68)	63 (54–69)	0.080	60 (52–68)	63 (53–69)	**0.015**
Sex, male, *N* (%)	464 (66.5%)	114 (56.7%)	**0.011**	360 (69.1%)	218 (57.7%)	**<0.001**
Hypertension, *N* (%)	542 (77.7%)	157 (78.1%)	0.890	399 (76.6%)	300 (79.4%)	0.322
Diabetes, *N* (%)	237 (34.0%)	73 (36.3%)	0.534	166 (31.9%)	144 (38.1%)	0.052
Dyslipidemia, *N* (%)	313 (44.8%)	99 (49.3%)	0.269	231 (44.3%)	181 (47.9%)	0.292
CAD, *N* (%)	96 (13.8%)	34 (16.9%)	0.261	60 (11.5%)	70 (18.5%)	**0.003**
Smoking, *N* (%)	304 (43.6%)	86 (42.8%)	0.847	241 (46.3%)	149 (39.4%)	**0.041**
Drinking, *N* (%)	228 (32.7%)	74 (36.8%)	0.272	189 (36.3%)	113 (29.9%)	**0.046**
IVT, *N* (%)	15 (2.1%)	6 (3.0%)	0.439	10 (1.9%)	11 (2.9%)	0.375
EVT, *N* (%)	22 (3.2%)	3 (1.5%)	0.328	17 (3.3%)	8 (2.1%)	0.412
Dual antiplatelets, *N* (%)	349 (50.0%)	84 (41.8%)	**0.045**	281 (53.9%)	152 (40.2%)	**<0.001**
**Severity of stenosis**, ***N*****(%)**			**<0.001**			**<0.001**
Mild	128 (18.3%)	20 (10.0%)		98 (18.8%)	50 (13.2%)	
Moderate	170 (24.4%)	42 (20.9%)		118 (22.6%)	94 (24.9%)	
Severe	154 (22.1%)	34 (16.9%)		127 (24.4%)	61 (16.1%)	
Occlusion	246 (35.2%)	105 (52.2%)		178 (34.2%)	173 (45.8%)	
**Number of stenosis**, ***N*****(%)**			0.821			0.610
1	191 (27.4%)	51 (25.4%)		137 (26.3%)	105 (27.8%)	
2	148 (21.2%)	42 (20.9%)		116 (22.3%)	74 (19.6%)	
≥ 3	359 (51.4%)	108 (53.7%)		268 (51.4%)	199 (52.6%)	
HT, *N* (%)	37 (5.3%)	15 (7.5%)	0.235	26 (5.0%)	26 (6.9%)	0.249
**ECASS II classification**, ***N*****(%)**			0.320			0.161
HI1, *N* (%)	9 (1.3%)	3 (1.5%)		6 (1.2%)	6 (1.6%)	
HI2, *N* (%)	15 (2.1%)	4 (2.0%)		13 (2.5%)	6 (1.6%)	
PH1, *N* (%)	5 (0.7%)	5 (2.5%)		3 (0.6%)	7 (1.9%)	
PH2, *N* (%)	8 (1.1%)	3 (1.5%)		4 (0.8%)	7 (1.9%)	
Moderate–severe CED, *N* (%)	43 (6.2%)	44 (21.9%)	**<0.001**	39 (7.5%)	48 (12.7%)	**0.012**
SBP (mmHg)	144 (132–158)	147 (134–160)	0.208	143 (132–156)	146 (134–160)	0.102
DBP (mmHg)	84 (76–93)	85 (76–92)	0.861	84 (76–93)	85 (76–92)	0.986
WBC (×10^9^/L)	6.70 (5.50–8.10)	7.70 (6.30–9.75)	**<0.001**	6.70 (5.47–8.10)	7.20 (5.90–8.70)	**<0.001**
Plt (×10^9^/L)	205.00 (169.00–246.00)	218.00 (171.75–259.50)	0.06	204.00 (164.50–244.00)	211.50 (176.00–256.25)	**0.021**
Neutrophil (×10^9^/L)	4.30 (3.30–5.40)	5.30 (4.00–7.00)	**<0.001**	4.20 (3.20–5.30)	4.80 (3.80–6.40)	**<0.001**
Lymphocyte (×10^9^/L)	1.60 (1.20–2.00)	1.40 (1.10–1.90)	**0.003**	1.60 (1.20–2.00)	1.50 (1.20–1.90)	**0.010**
NLR	2.63 (1.93–3.64)	3.53 (2.43–5.25)	**<0.001**	2.60 (1.88–3.54)	3.18 (2.25–4.53)	**<0.001**
TC (mmol/L)	4.37 (3.54–5.16)	4.42 (3.74–5.25)	0.157	4.34 (3.54–5.14)	4.45 (3.63–5.20)	0.168
TG (mmol/L)	1.57 (1.18–2.11)	1.54 (1.19–2.20)	0.991	1.58 (1.18–2.11)	1.54 (1.20–2.17)	0.924
HDL (mmol/L)	1.00 (0.85–1.18)	1.02 (0.89–1.23)	0.131	0.99 (0.84–1.17)	1.02 (0.88–1.24)	**0.045**
LDL (mmol/L)	2.66 (2.10–3.29)	2.75 (2.16–3.35)	0.143	2.63 (2.10–3.27)	2.76 (2.13–3.38)	0.133
FBG (mmol/L)	5.56 (4.91–7.17)	6.15 (5.19–7.96)	**<0.001**	5.54 (4.91–6.99)	5.95 (5.07–7.82)	**0.002**
UA (μmol/L)	315.70 (261.45–381.72)	290.60 (230.95–365.00)	**0.002**	318.50 (271.10–383.19)	300.39 (234.57–370.55)	**0.003**
Hcy (μmol/L)	13.45 (11.04–16.41)	12.59 (11.11–16.51)	0.578	13.43 (11.12–16.48)	13.27 (11.01–16.36)	0.445
Fib (g/L)	3.20 (2.74–3.89)	3.69 (2.96–4.39)	**<0.001**	3.15 (2.71–3.86)	3.45 (2.90–4.25)	**<0.001**

*Bold values denote statistical significance at the P < 0.05 level. NIHSS, the National Institutes of Health Stroke Scale; mRS, modified Rankin Scale; sICAS, symptomatic intracranial atherosclerotic stenosis; CAD, coronary artery disease; IVT, intravenous thrombolysis; EVT, endovascular therapy; HT, hemorrhagic transformation; ECASS II, European Cooperative Acute Stroke Study II; HI, hemorrhagic infarction; PH, parenchymal hematoma; CED, cerebral edema; SBP, systolic blood pressure; DBP, diastolic blood pressure; WBC, white blood cell; Plt, platelet; NLR, neutrophil-to-lymphocyte ratio; TC, total cholesterol; TG, triglyceride; HDL, high-density lipoprotein; LDL, low-density lipoprotein; FBG, fasting blood glucose; UA, uric acid; Hcy, homocysteine; Fib, fibrinogen*.

**Table 3:** Multivariate logistic regression analysis of the relationship between NLR and the initial stroke severity of sICAS.

	**Model 1**				**Model 2**		
	***P*-value**	**OR**	**95% CI**		***P*-value**	**OR**	**95% CI**
Age (years)	0.227	1.011	0.993–1.030	Age (≥61 years)	0.063	1.461	0.980–2.177
Sex (male vs. female)	**0.028**	0.634	0.423–0.951	Sex (male vs. female)	0.073	0.689	0.459–1.035
NLR	**0.005**	1.132	1.038–1.234	**NLR tertiles**			
WBC (×10^9^/L)	**0.020**	1.108	1.016–1.208	Tertile1 (<2.231)	Reference		
UA (μmol/L)	0.209	0.999	0.997–1.001	Tertile2 (2.231–3.533)	**0.023**	1.851	1.089–3.145
FBG (mmol/L)	0.269	1.040	0.970–1.115	Tertile3 (≥3.533)	**<0.001**	2.736	1.590–4.708
Fib (g/L)	0.101	1.082	0.985–1.190	WBC (≥6.9 × 10^9^/L)	0.237	1.290	0.846–1.966
Dual antiplatelets	0.676	1.085	0.739–1.594	UA (≥311.2 μmol/L)	0.290	0.806	0.541–1.201
**Severity of stenosis**				FBG (≥5.69 mmol/L)	**0.008**	1.700	1.147–2.521
Mild	Reference			Fib (≥3.28 g/L)	**0.001**	2.048	1.357–3.091
Moderate	0.636	1.180	0.594–2.345	Dual antiplatelets	0.604	1.109	0.751–1.638
Severe	0.561	1.232	0.610–2.487	**Severity of stenosis**			
Occlusion	**0.013**	2.183	1.183–4.029	Mild	Reference		
Moderate–severe CED	**<0.001**	3.105	1.785–5.403	Moderate	0.723	1.136	0.561–2.301
				Severe	0.594	1.213	0.596–2.470
				Occlusion	**0.017**	2.140	1.143–4.006
				Moderate–severe CED	**<0.001**	3.095	1.765–5.426

*Bold values denote statistical significance at the P < 0.05 level. NLR, neutrophil-to-lymphocyte ratio; sICAS, symptomatic intracranial atherosclerotic stenosis; WBC, white blood cell; FBG, fasting blood glucose; UA, uric acid; Fib, fibrinogen; CED, cerebral edema; OR, odds ratio; CI, confidence interval*.

### Association Between NLR and Short-Term Prognosis in sICAS Patients

Among all participants, 521 (58.0%) had a favorable short-term functional outcome, and 378 (42.0%) had an unfavorable short-term functional outcome as mentioned before. In univariate analysis, age, sex, histories of CAD, smoking, and drinking, dual antiplatelet therapy, stenosis severity, moderate-severe CED, and levels of WBC, Plt, neutrophils, lymphocytes, NLR, HDL, FBG, UA, and Fib were significantly correlated with short-term prognosis in sICAS patients (as shown in Table 2). NLR levels were statistically elevated in patients with unfavorable outcomes (median, 3.18; IQR, 2.25–4.53) compared with those with favorable outcomes (median, 2.60; IQR, 1.88–3.54; *P* < 0.001) (as shown in Table 2 and Figure 3B). Moreover, age, the proportions of female gender, occlusion, and moderate-severe CED, the prevalence of CAD, and levels of WBC, Plt, neutrophil, HDL, FBG, and Fib in the unfavorable outcome group were higher than those in the favorable outcome group, whereas the proportion of dual antiplatelet therapy, the frequencies of smoking and drinking, and the levels of lymphocyte and UA were lower in the unfavorable outcome group (as shown in Table 2). Multivariate logistic regression analysis showed that admission NLR levels were significantly correlated with unfavorable short-term prognosis (OR, 1.102; 95% CI, 1.017–1.195; *P* = 0.018) in Model 1 and the highest NLR tertile was positively associated with unfavorable short-term prognosis (OR, 2.165; 95% CI, 1.416–3.311; *P* < 0.001) compared with the lowest NLR tertile in Model 2 after adjusting for age, sex, CAD, smoking, drinking, dual antiplatelet therapy, severity of stenosis, moderate-severe CED, WBC, Plt, HDL, FBG, UA, and Fib (as shown in Table 4).

**Table 4:** Multivariate logistic regression analysis of the relationship between NLR and the short-term prognosis of sICAS.

	**Model 1**				**Model 2**		
	***P*-value**	**OR**	**95% CI**		***P*-value**	**OR**	**95% CI**
Age (years)	0.286	1.008	0.993–1.024	Age (>61 years)	0.146	1.277	0.919–1.775
Sex (male vs. female)	0.107	0.704	0.459–1.079	Sex (male vs. female)	0.092	0.686	0.443–1.064
CAD	**0.007**	1.816	1.180–2.796	CAD	**0.007**	1.828	1.178–2.839
Smoking	0.376	1.214	0.790–1.864	Smoking	0.212	1.324	0.852–2.057
Drinking	0.229	0.772	0.507–1.177	Drinking	0.167	0.736	0.477–1.136
NLR	**0.018**	1.102	1.017–1.195	**NLR tertiles**			
WBC (×10^9^/L)	0.257	1.050	0.965–1.143	Tertile1 (<2.231)	Reference		
Plt (×10^9^/L)	0.842	1.000	0.998–1.003	Tertile2 (2.231–3.533)	0.111	1.374	0.929–2.033
HDL (mmol/L)	0.835	0.954	0.614–1.483	Tertile3 (≥3.533)	**<0.001**	2.165	1.416–3.311
UA (μmol/L)	**0.030**	0.998	0.997–1.000	WBC (≥6.9 × 10^9^/L)	0.476	1.139	0.796–1.632
FBG (mmol/L)	0.054	1.062	0.999–1.128	Plt (≥207 × 10^9^/L)	0.948	1.011	0.722–1.416
Fib (g/L)	0.520	1.030	0.942–1.127	HDL (≥1 mmol/L)	0.985	1.003	0.720–1.398
Dual antiplatelets	**0.037**	0.717	0.525–0.980	UA (≥311.2 μmol/L)	0.094	0.756	0.546–1.048
**Severity of stenosis**				FBG (≥5.685 mmol/L)	**0.044**	1.390	1.008–1.915
Mild	Reference			Fib (≥3.28 g/L)	**0.019**	1.482	1.067–2.059
Moderate	0.342	1.273	0.774–2.094	Dual antiplatelets	0.059	0.735	0.533–1.012
Severe	0.482	0.829	0.490–1.400	**Severity of stenosis**			
Occlusion	**0.020**	1.727	1.090–2.736	Mild	Reference		
Moderate–severe CED	0.856	1.051	0.615–1.797	Moderate	0.472	1.208	0.722–2.020
				Severe	0.352	0.776	0.454–1.325
				Occlusion	**0.032**	1.674	1.045–2.683
				Moderate–severe CED	0.968	1.011	0.585–1.746

*Bold values denote statistical significance at the P < 0.05 level. NLR, neutrophil-to-lymphocyte ratio; sICAS, symptomatic intracranial atherosclerotic stenosis; CAD, coronary artery disease; WBC, white blood cell; Plt, platelet; HDL, high-density lipoprotein; FBG, fasting blood glucose; UA, uric acid; Fib, fibrinogen; CED, cerebral edema; OR, odds ratio; CI, confidence interval*.

### Predictive Values of NLR for the Severity and Short-Term Prognosis of sICAS

**Figure 4:**
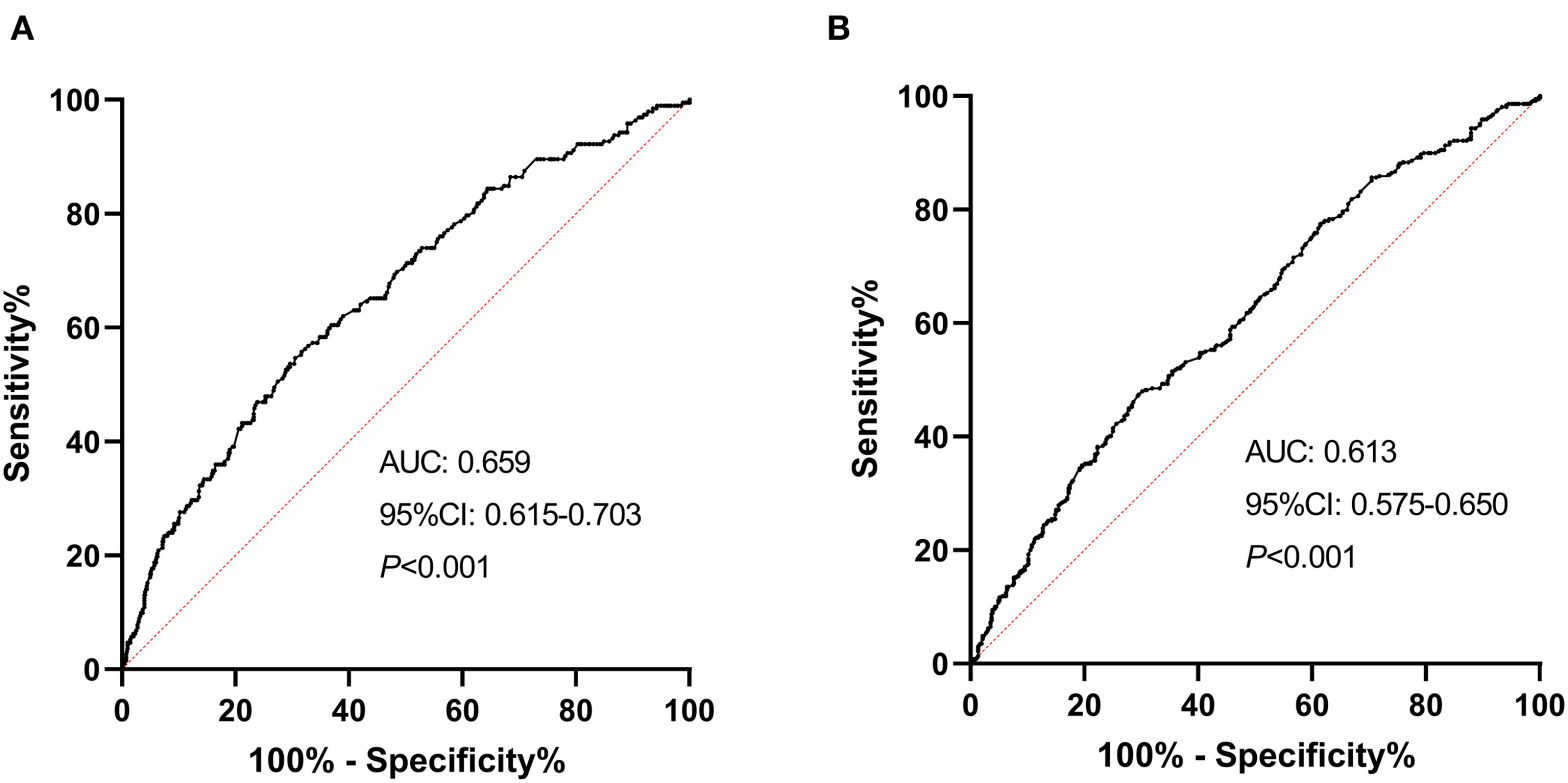
ROC curves for the initial stroke severity and short-term prognosis of sICAS with NLR. **(A)** ROC curve for the initial stroke severity of sICAS with NLR (severe stroke vs. mild controls). **(B)** ROC curve for the short-term prognosis of sICAS with NLR (unfavorable outcome vs. favorable outcome). ROC, receiver operating characteristic; sICAS, symptomatic intracranial atherosclerotic stenosis; AUC, area under the curve; NLR, neutrophil-to-lymphocyte ratio; CI, confidence interval.

Receiver operating characteristic curve analysis was conducted to determine the predictability of NLR with the results illustrated in Figure 4. Area under the curve values for discriminating stroke severity and short-term prognosis were 0.659 (95% CI, 0.615–0.703; *P* < 0.001) and 0.613 (95% CI, 0.575–0.650; *P* < 0.001), respectively, and the optimal cut-off values of NLR were 3.33 (specificity, 69.49%; sensitivity, 54.87%) for stroke severity and 3.31 (specificity, 70.45%; sensitivity, 47.43%) for short-term prognosis (as shown in Figure 4).

### Predictive Models of the Severity and Short-Term Outcome of sICAS

**Figure 5:**
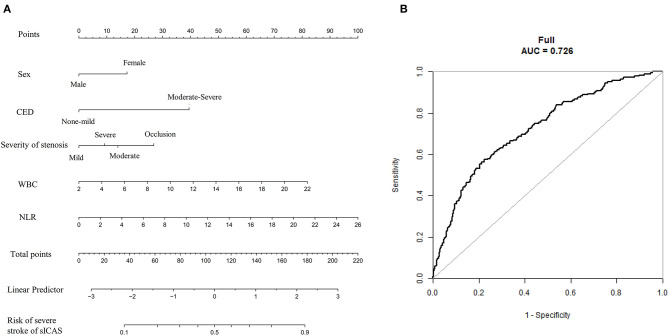
The nomogram for predicting the severity of sICAS patients. **(A)** The nomogram for predicting the risk of severe neurological deficit of sICAS patients. **(B)** The ROC curve of the nomogram. The predictors were chosen based on the results of multivariate logistic regression in Model 1. Each selected factor was shown by a line in the nomograms. sICAS, symptomatic intracranial atherosclerotic stenosis; CED, cerebral edema; WBC, white blood cell; NLR, neutrophil-to-lymphocyte ratio; ROC, receiver operating characteristic; AUC, area under the curve.

**Figure 6:**
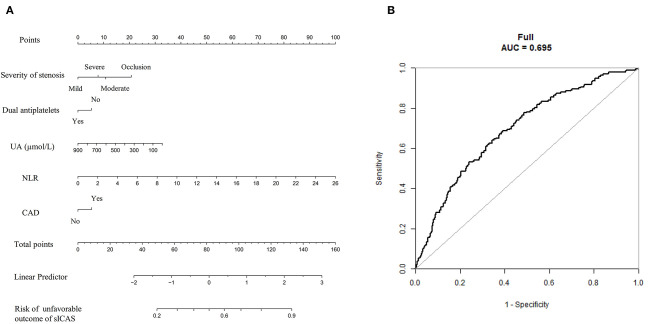
The nomogram for predicting the short-term prognosis of sICAS patients. **(A)** The nomogram for predicting the risk of unfavorable short-term outcome of sICAS patients. **(B)** The ROC curve of the nomogram. The predictors were chosen based on the results of multivariate logistic regression in Model 1. Each selected factor was shown by a line in the nomograms. sICAS, symptomatic intracranial atherosclerotic stenosis; NLR, neutrophil-to-lymphocyte ratio; UA, uric acid; CAD, coronary artery disease; ROC, receiver operating characteristic; AUC, area under the curve.

To establish a more direct correlation of NLR with sICAS, we constructed nomograms using the variables identified by multivariate logistic regression analysis. We chose the independent risk factors identified in Model 1. The selected factors were shown by lines in the nomograms. Higher nomogram total scores indicated a higher risk of severe neurological deficit or unfavorable short-term outcome of sICAS patients. Figures 5A, 6A showed the nomograms for predicting the severity and short-term prognosis of sICAS patients. Figures 5B, 6B showed the performance of the nomograms. Supplementary Figures 1A–D showed the nomograms using the variables identified by multivariate logistic regression in Model 2 as well as their performances.

## Discussion

In the present study, we demonstrated that elevated admission NLR was significantly correlated with initial stroke severity and poor short-term prognosis in sICAS patients. The statistical significance remained after adjustment for clinical and laboratory variables when NLR was 3.533 or more. Moreover, we demonstrated that NLR had good predictive power for the early severity and short-term prognosis of sICAS.

Our results are in accordance with prior studies relating to NLR and ischemic stroke. Researchers have reported that NLR was independently associated with stroke severity, hemorrhagic complications, poor short-term and long-term functional outcomes, high mortality rates, and stroke-associated pneumonia in acute cerebral ischemia (23–26). In patients with AIS attributable to LAA, high NLR was associated with increased risks for HT after LAA (27). In our study, we also observed that NLR values were higher in sICAS patients with HT, but HT was not a risk factor for the severity and prognosis of sICAS. More recently, a study in Portugal found that increased NLR was linked to the greater severity of CED, early neurological deterioration (END), and worse long-term prognosis in AIS patients undergoing reperfusion therapy (21). However, the predictive role of NLR in the prognosis of ischemic stroke due to ICAS has not been defined before. Our study revealed a similar role of NLR; that is, NLR was an early predictor of the early stroke severity and poor short-term outcome in patients with AIS attributed to ICAS, independent of other risk factors including CED, whereas the underlying mechanisms remain unclear.

Intracranial atherosclerotic stenosis is one of the most predominant causes of ischemic stroke, with a higher prevalence in Asians (3). Prior studies have demonstrated that traditional cardiovascular risk factors, metabolic syndrome, and unhealthy lifestyles are crucial risk factors associated with ICAS, accounting for endothelial dysfunction and increased permeability, thereby initiating a series of inflammatory reactions, which have been considered to be implicated in the pathogenesis of atherosclerosis and might play a critical role in the development of ICAS and plaque destabilization (8, 28). Although there are various risk factors and predictive models for ICAS, more convenient and easily accessible biomarkers are required to identify high-risk patients. Neutrophil-to-lymphocyte ratio, a biomarker indicating systemic inflammatory status, has been shown to be related to intracranial atherosclerosis. A study by Chung et al. (29) revealed that high NLR was correlated with intracranial atherosclerosis (OR= 1.87; 95% CI, 1.15–3.06; *P* = 0.01) in healthy individuals, consistent with another study by Nam et al. (15) indicating that NLR could be a predictor for the prevalence and burdens of ICAS. However, few studies revealed the independent predictive values of NLR in the prognosis of sICAS. Our prior study has determined the association between leukocyte levels and the prognosis of sICAS, whereas NLR might be a more accurate biomarker reflecting the balance of neutrophils and lymphocytes. Therefore, we examined whether NLR levels correlate with the early stroke severity and prognosis of sICAS and indicated that NLR could be employed as a good predictor with respect to the severity and short-term prognosis of sICAS.

Since NLR represents the ratio of neutrophils to lymphocytes, both neutrophilia and lymphopenia could be associated with increased NLR levels, providing clues to the possible underlying mechanisms. A growing body of evidence suggests that elevated circulating neutrophils were related to stroke severity, infarction volume, hemorrhagic complications, and poor outcomes in ischemic stroke (24, 30, 31). When stroke events occur, blood neutrophils could be rapidly recruited to areas of ischemic brain, release matrix metalloproteinases-9 (MMP-9), which could degrade tight junction proteins and basal lamina type IV collagen, increasing the blood-brain barrier (BBB) permeability, thereby resulting in BBB disruption and tissue damage, which is accompanied by edema and hemorrhage (32–35). Besides, an experimental study demonstrated that accumulated neutrophils could impair vascular remodeling during stroke recovery via producing neutrophil extracellular traps (NETs) (36). The expanded neutrophils after ischemic stroke might result from hematopoietic stem cell activation and a hematopoiesis bias mediated by increased sympathetic tone (37). Additionally, neutrophils are implicated in the development and progression of atherosclerosis. Neutrophils could be recruited and activated on the vascular wall, secreting granule proteins and limiting the use of nitric oxide, thus leading to endothelial dysfunction and subsequent atherosclerosis (38). Also, neutrophils could induce macrophages to release cytokines and activate T helper 17 (TH17) cells via releasing NETs, further amplifying pro-inflammatory responses and causing atherosclerotic plaque destabilization and rupture (39, 40).

However, the exact role of lymphocytes in atherosclerosis and ischemic stroke has not been fully elucidated. A study by Kim et al. (41) demonstrated the associations between reduced lymphocyte counts with END and unfavorable long-term prognosis in cerebral ischemia, implying that lymphocytes might be a protector for brain injury. It could be supported by another study, which reported that a lower admission lymphocyte-to-monocyte ratio (LMR) was an independent predictor for greater stroke severity and worse 3-month functional outcomes of acute cerebral infarction (42). Since the initial severity of stroke paralleled a more robust stress response, lymphopenia has been suspected to be mechanistically mediated by stress-induced cortisol production and sympathetic tone after stroke, which could further induce redistribution of lymphocytes and lymphocyte apoptosis (43, 44). Another possible mechanism lies in the protective role of regulatory lymphocytes in stroke via secreting interleukin-10 (IL-10), a critical anti-inflammatory and neuroprotective cytokine modulating post-stroke neuroinflammation (45, 46). Collectively, both inflammation and immune responses have considerable influence on the pathogenesis of ischemic stroke.

Early risk assessment is essential in determining the appropriate treatment and improving long-term life quality in patients with sICAS. Hence, it is critical to seek early predictors of sICAS to identify patients at high risk of severe stroke and unfavorable outcomes for early clinical intervention. Admission NLR, as an easy and straightforward biomarker, has the potential to help clinicians make decisions early and accurately. Our findings suggested that high NLR levels were correlated with severe stroke and poor prognosis in AIS patients due to ICAS, highlighting a promising potential therapeutic strategy for sICAS by reducing the ratio, which requires further investigations.

## Limitations

There are several limitations in this study that should be noted. First, a selection bias might exist since it was a retrospective study with participants recruited from a single stroke center. Second, our data are descriptive, so associations do not necessarily imply causality. Further studies remain to be conducted to elucidate the causality between NLR with early severity and outcomes in AIS patients with ICAS. Third, NLR was only examined on admission, and longitudinal data were lacking. Otherwise, more precise and consistent information could be provided. Moreover, the functional outcomes were only assessed on the 14^th^ day after stroke onset, limiting the predictive values of NLR for long-term prognosis in ICAS-related stroke. Forthcoming studies should be undertaken to examine whether NLR levels could estimate stroke recurrence or dementia after sICAS.

## Conclusions

This study has demonstrated that elevated admission NLR was independently correlated with severe stroke and poor functional outcome of sICAS and served as a useful parameter for early prediction of severity and short-term prognosis of sICAS. Therefore, NLR might be an essential and practical prognostic marker in patients with sICAS, helping us identify a target group for preventive therapies.

## Data Availability Statement

The original contributions presented in the study are included in the article/Supplementary Material, further inquiries can be directed to the corresponding author/s.

## Ethics Statement

The studies involving human participants were reviewed and approved by Medical Ethics Committee of Xiangya Hospital, Central South University, China. The patients/participants provided their written informed consent to participate in this study.

## Author Contributions

JX and FY conceptualized and designed the study. YY drafted the manuscript and interpreted the data. FY analyzed the data and revised the manuscript. YL, XF, DL, MW, XL, QH, ZL, LZ, TZ, and RT collected the data. All authors contributed to the article and approved the final version to be published.

## Conflict of Interest

The authors declare that the research was conducted in the absence of any commercial or financial relationships that could be construed as a potential conflict of interest.

## Publisher's Note

All claims expressed in this article are solely those of the authors and do not necessarily represent those of their affiliated organizations, or those of the publisher, the editors and the reviewers. Any product that may be evaluated in this article, or claim that may be made by its manufacturer, is not guaranteed or endorsed by the publisher.
