# Supplementary material for: Neutrophil-to-Lymphocyte Ratio as a Predictive Biomarker for Stroke Severity and Short-Term Prognosis in Acute Ischemic Stroke With Intracranial Atherosclerotic Stenosis
Source: Front Neurol. 2021 Jul 29;12:705949. doi: 10.3389/fneur.2021.705949 (PMC8360230; doi:10.3389/fneur.2021.705949)
Supplement: Supplementary file 1 [file Data_Sheet_1.docx]

Figure S1


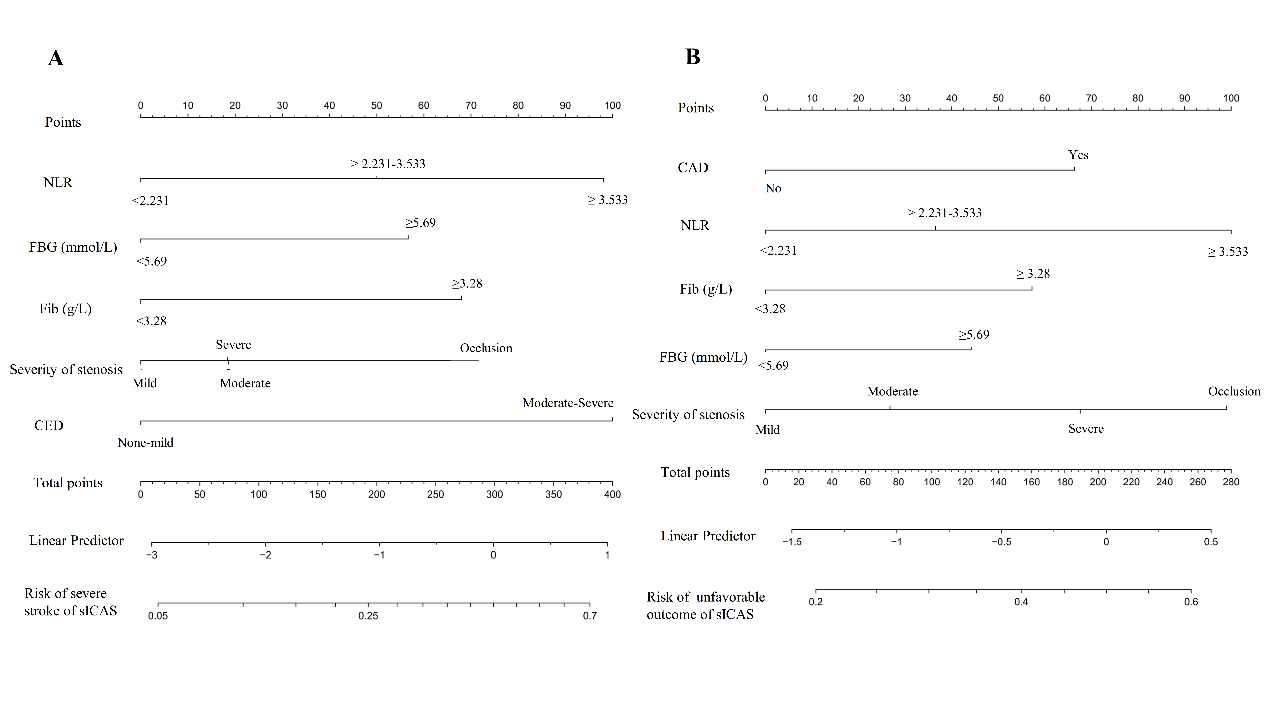


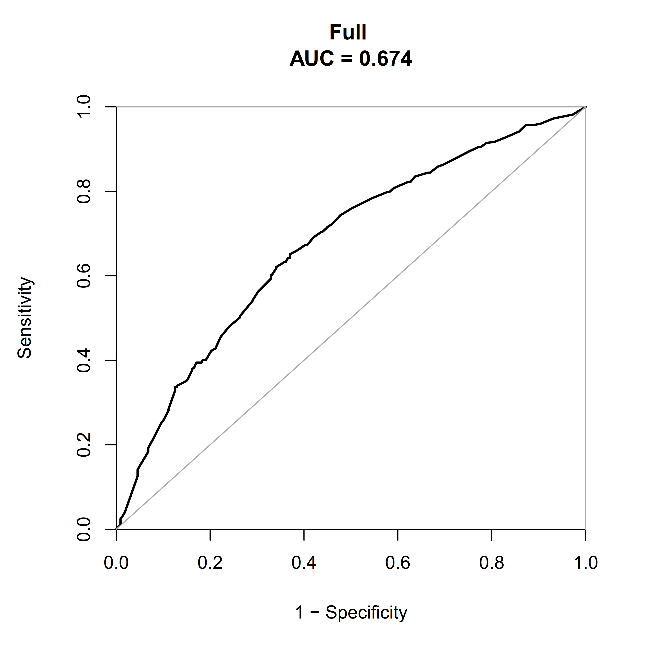

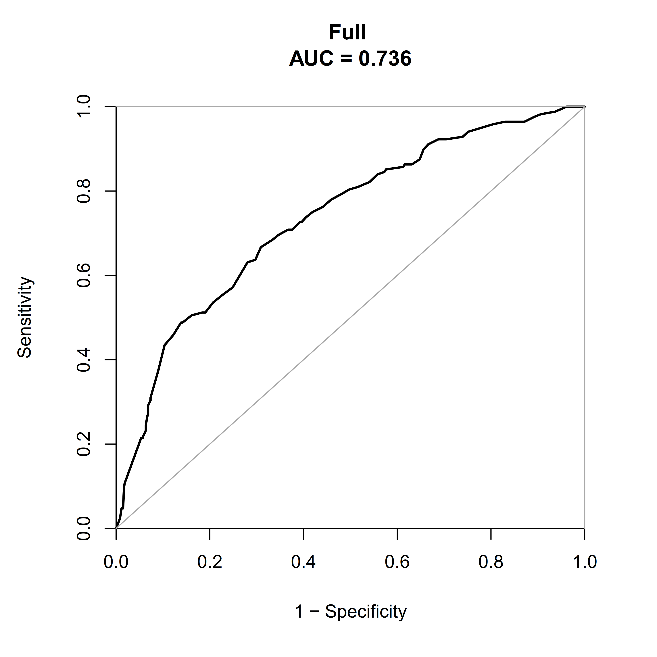


**D**C

**C**C

The nomograms for predicting the severity and short-term prognosis of sICAS patients. **(A)** The nomogram for predicting the risk of severe stroke symptoms of sICAS patients. **(B)** The nomogram for predicting the unfavorable short-term outcome of sICAS patients. **(C)** The ROC curve of the nomogram in (**A**). **(D)** The ROC curve of the nomogram in (**B**). The predictors were chosen based on the results of multivariate logistic regression in Model 2. Each selected factor was shown by a line in the nomograms. sICAS, symptomatic intracranial atherosclerotic stenosis; CED, cerebral edema; NLR, neutrophil-to-lymphocyte ratio; FBG, fasting blood glucose; Fib, fibrinogen; CAD, coronary artery disease; ROC, receiver operating characteristic; AUC, area under the curve.
